# RNA Sequencing Dataset of *Drosophila* Nociceptor Translatomic Response to Injury

**DOI:** 10.3390/data10020011

**Published:** 2025-01-21

**Authors:** Christine M. Hale, Kyle J. Beauchemin, Courtney L. Brann, Julie K. Moulton, Ramaz Geguchadze, Benjamin J. Harrison, Geoffrey K. Ganter

**Affiliations:** 1 College of Arts and Sciences, University of New England, 11 Hills Beach Road, Biddeford, ME 04103, USA; 2 College of Osteopathic Medicine, University of New England, 11 Hills Beach Road, Biddeford, ME 04103, USA

**Keywords:** allodynia, hyperalgesia, RNAseq, nociceptor, pain, sensitization

## Abstract

To prepare to address the mechanisms of injury-induced nociceptor sensitization, we sequenced the translatome of the nociceptors of injured *Drososophila* larvae and those of uninjured larvae. Third-instar larvae expressing a green fluorescent protein (GFP)-tagged ribosomal subunit specifically in Class 4 dendritic arborization neurons, recognized as pickpocket-expressing primary nociceptors, via the GAL4/UAS method, were injured by ultraviolet light or sham-injured. Larvae were subjected to translating ribosome affinity purification for the GFP tag and nociceptor-specific ribosome-bound RNA was sequenced.

## 1. Summary

Uncontrolled pain places an enormous burden on human life. Currently, analgesic medications inadequately address the problem due to issues with efficacy, tolerance, side effects, and addiction liability, particularly in chronic pain conditions. Improved therapies for managing abnormal pain, including chronic pain, are needed. Chronic pain often involves nociceptor sensitization, and one way in which nociceptors become sensitized is by injury. The primary nociceptors in *Drosophila* larvae can be sensitized by injuring the adjacent epidermis with ultraviolet radiation. We previously showed that UV-induced sensitization requires activity of the Bone Morphogenetic Protein pathway, which is known to affect gene expression. To reveal translatomic changes in primary nociceptors caused by injury, we isolated translating mRNAs from the nociceptors of injured and uninjured animals using the Translating Ribosome Affinity Purification method and compared the resulting sequences. Because over 75% of all identified human disease genes have orthologs in the fly genome, the *Drosophila* model system has the capacity to rapidly identify valuable targets for novel analgesic therapies.

## 2. Data Description

### 2.1. Background and Summary

After injury, healing is promoted by nociceptive sensitization, an increase in pain sensitivity in and around the damaged tissue. However, sensitization can also perpetuate abnormal pain states like chronic pain [1]. Nociceptive sensitization, including allodynia and hyperalgesia, can be induced by ultraviolet (UV) light injury in the larval fruit fly, revealing novel regulators of this process [2,3]. Although in this model the epidermis is severely compromised by ultraviolet irradiation, nociceptor morphology appears to remain intact. While others [4,5] have explored nociceptor gene expression using microarray approaches and yielded important information about stimulus transduction, to our knowledge, there has been no prior analysis of the nociceptor-specific translatomic consequences of UV injury in *Drosophila*. We expect that this effort will reveal events in the primary nociceptor that lead to nociceptive sensitization.

**Figure 1.**
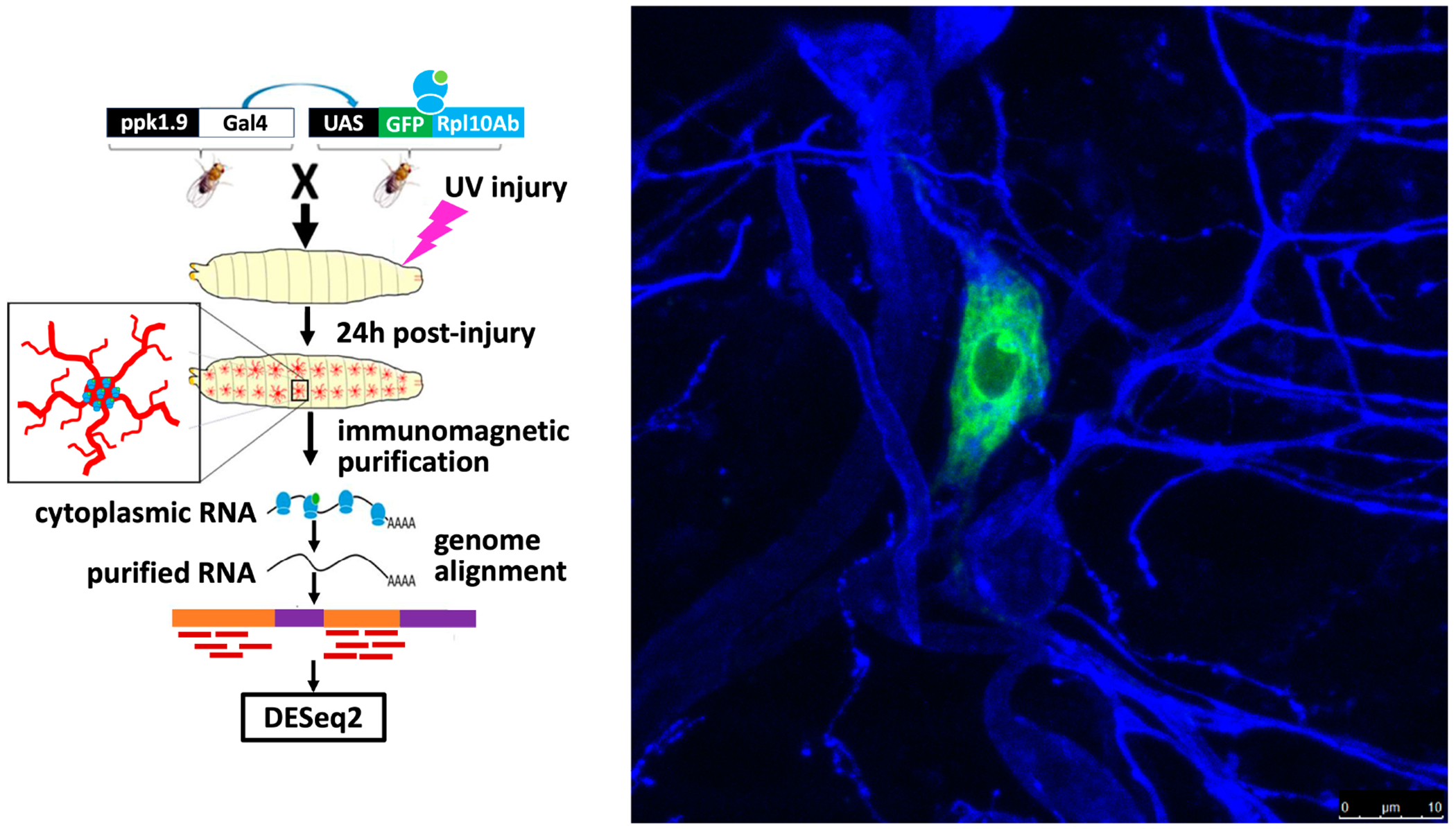
Translating Ribosomal Affinity Purification (TRAP) isolation of nociceptor RNA workflow. **Left**: Third-instar *Drosophila* larvae expressing UAS-GFP-RpL10Ab within their nociceptors (**right**) underwent UV injury and were given 24 h to recover, flash frozen, and then homogenized to undergo immunomagnetic purification of nociceptor GFP-tagged ribosomes. RNA sequencing vendor GENEWIZ^®^ carried out sequencing, mapped reads to the *Drosophila* reference genome, and determined gene hit counts. **Right**: Nociceptor expressing GFP-tagged ribosomal subunit (green). Adjacent neurons visualized with anti-HRP (blue).

Using the Translating Ribosome Affinity Purification approach (see Figure 1), we isolated mRNA from the primary nociceptors of third-instar *Drosophila* larvae 24 hours after UV injury, at which point they experience peak allodynia [2,6,7]. We hypothesized that transcriptional/translational responses to injury lead to the process of nociceptive sensitization and/or recovery from sensitization. Building on our prior research in nociceptive sensitization using fly larvae, we believe that further investigation into the mechanisms of its development and recovery will deepen our understanding of the complete nociceptive sensitization mechanism and reveal new targets for chronic pain drug development.

### 2.2. Technical Validation

Pairwise, Dispersion, and Principal Component Analysis

**Figure 2.**
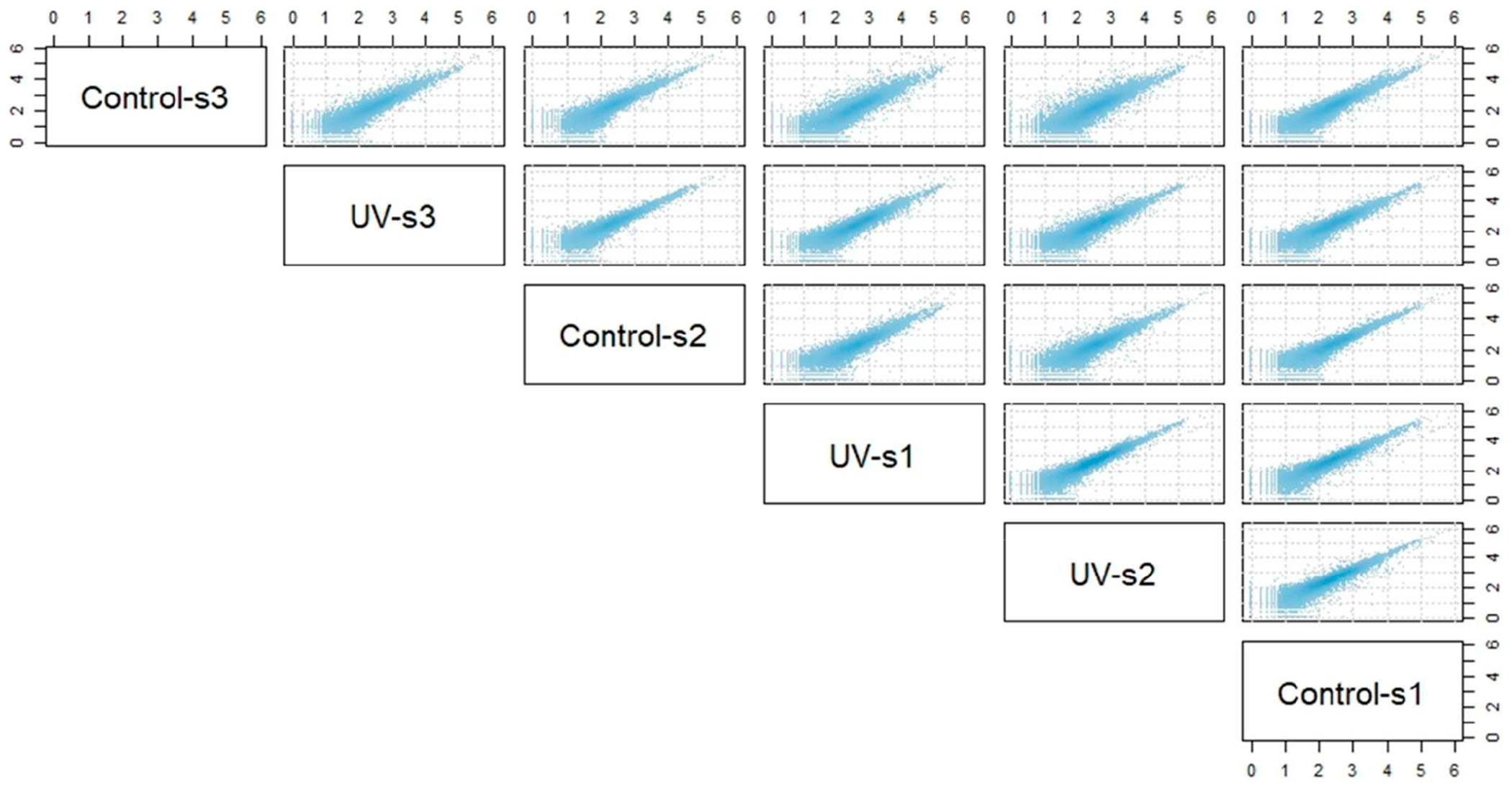
Pairwise scatterplots revealing intra-/inter-sample relationships. Sample gene counts from nociceptor transcripts were log10-transformed in R and were analyzed for quality and inter-/intra-relationships by pairwise scatterplots of all samples in both groups (Control vs. UV-injured). Each dot within the scatterplot represents a gene, and the mean expression of that gene between the two samples shown by its x-y coordinate placement. n = 3 (pooled samples)/group (Control vs. UV-injured).

**Figure 3.**
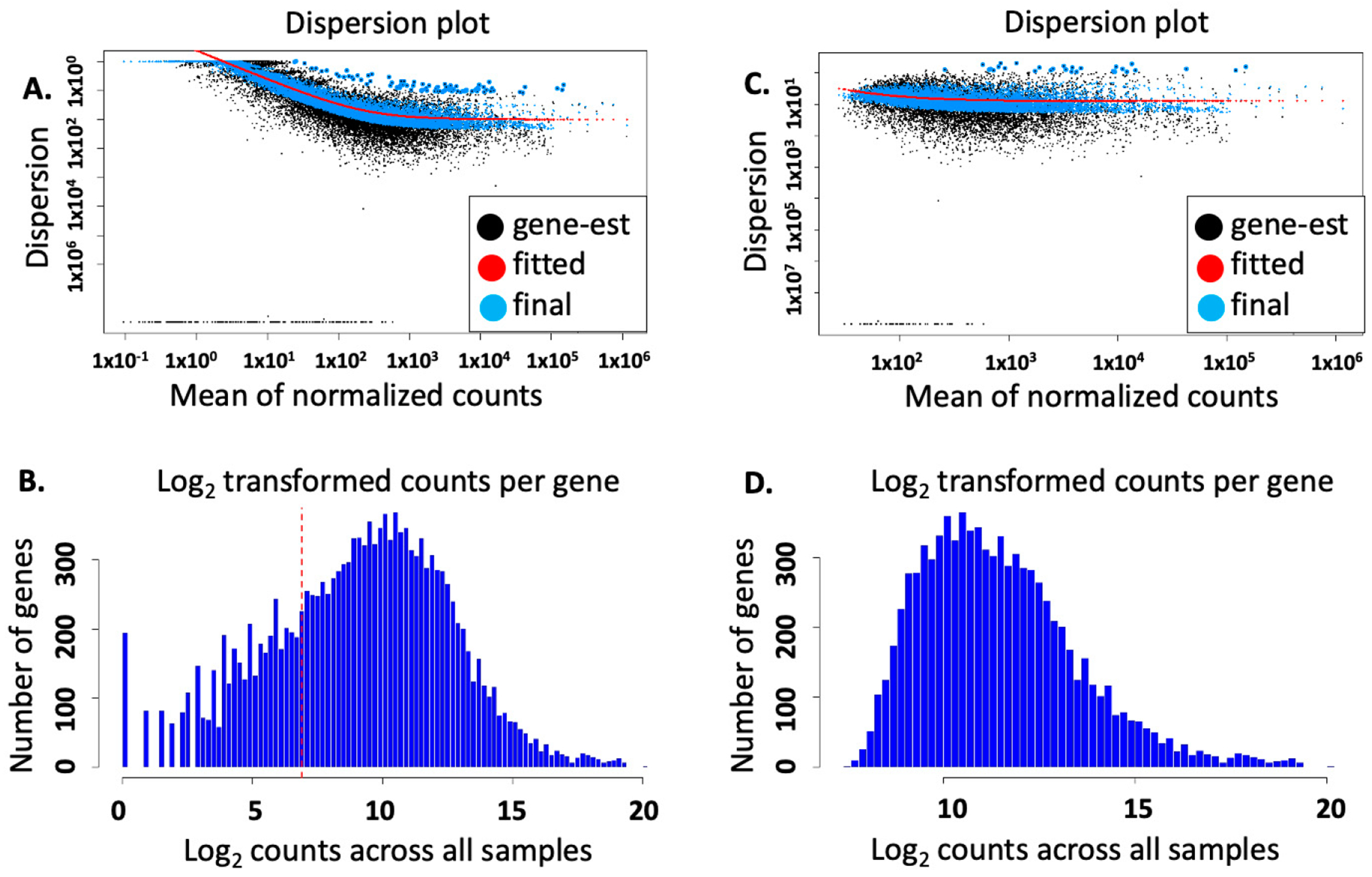
Dispersion and histogram plots visualizing sample quality and noise within the data. (**A**) Dispersion plot of the mean of the normalized counts was plotted using DESeq2. The plot estimates dispersion or intra-sample variability in a gene’s expression within each condition group (Control vs. UV-injured). Interestingly, analyses showed a high number of low-count features at the limit of the y-axis for estimated dispersion, and (**B**) the histogram of the log_2_ count data vs. number of genes expressed also displayed similar low-count features across all samples, even after removal of 0-count genes from the dataset. This indicated a robust degree of sensitivity due to the high sequencing depth (see Table 1) and may also indicate the detection of low-copy-number transcripts, long noncoding RNA’s, and additional species of transcript that can be the subject of future investigation. A conservative pre-threshold limit for counts across all samples (≥20 counts for each of the six samples (n = 3 pooled samples/condition (Control vs. Experimental)) was then established (visualized by the red dotted line). (**C**,**D**) Dispersion and histogram plots following the established threshold limit for counts across all samples to eliminate noise, showing the removal of low-count features.

The ‘estimateSizeFactors’ function in DESeq2 was carried out to control for differences in library sizes using the “median-of-ratios method” [8,9]. Inter-/intra-relationships among groups and sample quality were visualized by pairwise scatterplots of all samples in both groups (Figure 2) in R, using count data normalized by log10 transformation [10]. Read count distribution and the potential high magnitude of low read counts was investigated through visualization of a histogram of the sum of log10-transformed count data across all samples, also in R (Figure 3B,D) [11,12]. The DESeq2 function ‘estimateDispersions’ was then used to calculate dispersion estimates across genes for all samples and visualized with the DESeq2 dispersions plot (‘plotDispEsts’) (Figure 3A,C). After preliminary analysis of the count data for low expression, we set a custom threshold of at least 20 counts per six samples via the following code written in R and applied it to the dds object within the DESeq2 pipeline [12]: ‘keep <- rowSums(counts(dds, normalized=TRUE) >= 20) >= 6’ ‘dds <- dds[keep,]’.

**Figure 4.**
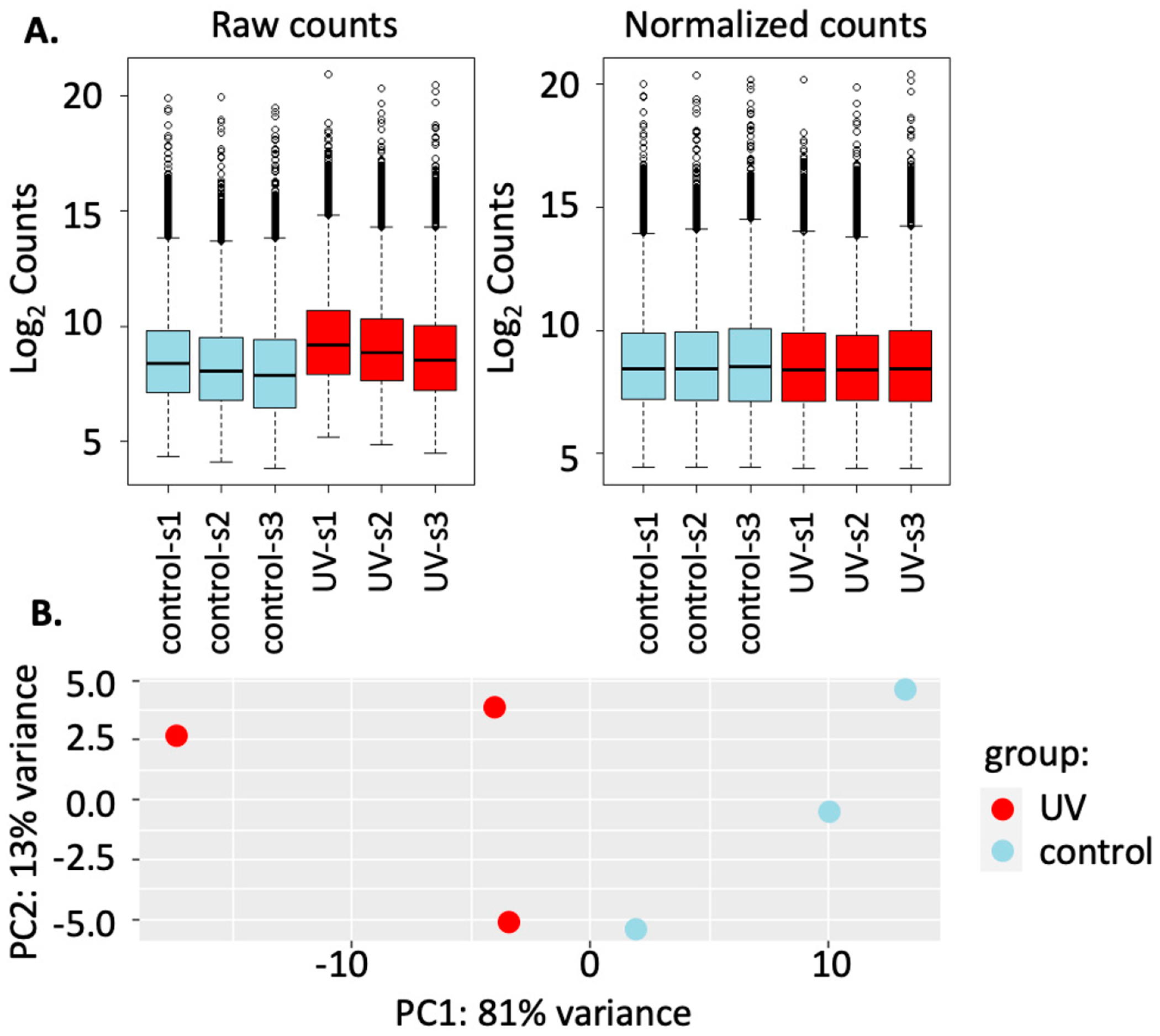
Analysis of normalization of gene count data across all samples and principal component analysis (PCA) of sample relationship post DESeq2 normalization in R. (**A**) Quality analysis was investigated by comparing bar plots of the raw (un-normalized) log_2_ count data across all samples to the normalized log_2_ count data after the DESeq2 function for differential gene expression analysis had been applied across all samples. (**B**) Clustering of samples per condition was visualized through a principal component analysis (PCA) plot, which breaks down the maximum levels of variation into components of the top 100 differentially expressed genes after regularized-logarithm transformation (rlog) in DESeq2.

After counts had been thresholded to eliminate sparsely expressed genes, the DESeq output was visualized for sample clustering analysis using DESeq-normalized data and the principal components plot function (Figure 4) found within the DESeq2 package [9]. Sequencing depth is indicated in Table 1.

## 3. Methods

### 3.1. Genetics

Flies were maintained in 6 oz stock bottles containing a sucrose–cornmeal–yeast medium. Bottles were stored in Percival Scientific Incubators with a 12 h light/12 h dark cycle and kept between 50–60% humidity and at a temperature of 25 °C. Incubators were set to an arbitrary dawn time of 9:00 A.M. Genotypes used in the experiments were prepared using the Gal4/UAS system [13] with the Gal4 driver line featuring the nociceptor-specific pickpocket promoter: ppk1.9-GAL4 (in w^1118^) [14–16]. UAS responder line was UAS-GFP-RpL10Ab [17] (in w*) (BDSC_42681), allowing for affinity purification [18].

### 3.2. Sensitizing Injury

Flies expressing the eGFP-tagged ribosomal subunit RpL10Ab specifically in nociceptors (approximately 70 cells per animal) were allowed to mate for 48 hours prior to the timed egg lay. After two days, the flies were placed in a tube containing solidified grape juice agar along one wall to encourage egg deposition. The egg-laying period was restricted to two hours, and then the adults were removed. Developmentally timed larvae were collected 4–5 days after egg laying and placed into a UV crosslinker (Spectrolinker XL-1000, Spectronics Corporation, Westbury, NY, USA), and the larvae were exposed to a dosage of UV-C between 12.0–18.0 mJ, monitored with a UV photometer (Spectroline XS-254 UV-C, Spectronics Corporation, Westbury, NY, USA). For mock-treated control animals, an identical protocol was performed, including putting the animals into the crosslinker, but without the actual delivery of UV. The larvae were placed in recovery vials for 24 h and then separated into 100 mg groups. Larvae were flash-frozen and stored in liquid nitrogen until analysis.

### 3.3. RNA Extraction, Sequencing, and Preliminary Analysis

Frozen larvae were homogenized, and homogenates underwent immunopurification of the eGFP-tagged ribosomes using magnetic beads (Invitrogen Dynabeads Antibody Coupling Kit, Carlsbad, CA, USA) bound to two anti-GFP antibodies (19C8 and 19F7, Memorial Sloan-Kettering Monoclonal Antibody Facility, New York, NY, USA). RNA was then isolated and purified from these eGFP-tagged ribosomes using a standard RNA isolation protocol (Machery-Nagel NucleoSpin RNA kit, Toronto, ON, Canada). RNA was then tested for quantity and purity with an Agilent Bioanalyzer, obtaining an RNA integrity number (RIN) ranging from 4.8 to 6.6. While RINs of 6 or greater are recommended by the RNA-sequencing vendor GENEWIZ^®^ (South Plainfield, NJ, USA), we judged the integrity to be sufficient, since RNA of insects typically scores lower than mammals, for which the RIN algorithm was developed [19]. RNA was stored at −80 °C before being shipped (7.5 to 13.5 nM) on dry ice to the vendor. GENEWIZ carried out mRNA sequencing via polyA selection with supplied RNA using Illumina HiSeq, PE 2x150 (150 bp paired end). GENEWIZ trimmed sequence reads via Trimmomatic v.0.36, mapped sequence reads to the Drosophila melanogaster BDGP6 reference genome via ENSEMBL using the STAR aligner v.2.5.2b, and determined gene hit counts (calculation of reads/gene/sample) using feature counts from the Subread package v.1.5.2. A total of six samples of customer-supplied RNA were used for RNA sequencing by the vendor: three control (mock-injured) samples and three experimental (UV-injured) samples, with each sample of RNA being derived from the 100 mg groups of prepared larvae that were pooled by condition. In the supplied deliverables by GENEWIZ were original text files of the unique gene hit counts (reads/gene) for each of these six samples. These individual counts files were used as inputs for further quality assessment.

### 3.4. Code Availability

The R code used to analyze and process the raw count data from control and UV-injured samples (Table 1) using DESeq2 package version on R version 4.0.3 is publicly available at https://github.com/gkganter/Hale-et-al.-DESeq2-Code/blob/main/TRAPseq_DESeq2_3_19_2022_1100am.R (accessed on 1 January 2025).

## Figures and Tables

**Table 1.** Sample identification and statistics. UV samples were derived from UV-injured late-third-instar larvae expressing GFP-tagged ribosomal subunit RPL10 under the control of the ppk promotor, thereby limiting its expression to the nociceptors. Control samples were identical to samples from UV-injured animals in every way except that no UV radiation was applied. Three biological replicates of each treatment made up each group. Sequencing depth is indicated in millions of reads per sample.

File Name	Sample Name	Organism	Reads/Sample	Tax ID	Breed
**SAMN39083405**	Control-s1	*Drosophila melanogaster*	111.6M	7227	ppk-gal4/uas-rpl10
**SAMN39083406**	Control-s2	*Drosophila melanogaster*	100.3M	7227	ppk-gal4/uas-rpl10
**SAMN39083407**	Control-s3	*Drosophila melanogaster*	112.5M	7227	ppk-gal4/uas-rpl10
**SAMN39083408**	UV-s1	*Drosophila melanogaster*	90.2M	7227	ppk-gal4/uas-rpl10
**SAMN39083409**	UV-s2	*Drosophila melanogaster*	107.3M	7227	ppk-gal4/uas-rpl10
**SAMN39083410**	UV-s3	*Drosophila melanogaster*	112.0M	7227	ppk-gal4/uas-rpl10

## Data Availability

The original data described in the study are openly available at https://www.ncbi.nlm.nih.gov/bioproject/PRJNA1056042 (accessed on 1 January 2025).
